# Delayed Traumatic Aortic Injury Caused by a Displaced Rib Fracture in an Elderly Patient: A Case Report

**DOI:** 10.7759/cureus.113924

**Published:** 2026-08-04

**Authors:** Shiro Fukuyama, Chihiro Iwanaga, Mitsuhito Sato, Kenshin Shimono, Shuhei Niiyama

**Affiliations:** 1 Department of Emergency and Intensive Care Medicine, Kagoshima University Hospital, Kagoshima, JPN

**Keywords:** delayed traumatic aortic injury, geriatric trauma, intra-aortic balloon occlusion, recurrent falls, rib fracture, thoracic endovascular aortic repair

## Abstract

Rib fractures are common injuries in elderly trauma patients and are typically managed conservatively with favorable outcomes. However, recurrent falls can cause displacement of initially stable rib fragments, leading to catastrophic internal organ injuries. We present a case of a male in his late 70s with a history of total aortic arch replacement who initially sustained an undisplaced left 10th rib fracture from a fall. Fifteen days later, a secondary fall at home resulted in severe displacement of the rib fragment, which directly penetrated the descending aorta. The patient presented in profound obstructive and hemorrhagic shock due to a tension hemothorax. Rapid stabilization was achieved using an intra-aortic balloon occlusion (IABO) catheter, followed by emergency thoracic endovascular aortic repair (TEVAR). Despite a complicated course with acute consumptive coagulopathy, the patient was successfully managed with a massive transfusion protocol and extubated on postoperative day 11. This case highlights the critical importance of recognizing the potential for delayed, fatal vascular complications from displaced rib fractures in elderly patients prone to recurrent falls.

## Introduction

Rib fractures are common injuries, and their incidence has been increasing year by year, particularly among the elderly [1]. In elderly males, approximately half of these injuries result from ground-level falls or falls from a standing height [2]. Fundamentally, conservative management is the cornerstone of treatment for these injuries. In cases of flail chest or multiple trauma, the mortality rate exceeds 10%. Furthermore, advanced age combined with three or more rib fractures is known to indicate a poor prognosis [3-5]; thus, the presence of rib fractures itself should be recognized as a risk factor for adverse events. However, the probability of an isolated rib fracture alone causing a fatal event is generally low [6]. In the vast majority of cases, these fractures are safely managed with adequate analgesia and close outpatient follow-up [7].

However, in patients with persistent frailty or a high risk of recurrent falls, the clinical trajectory can change dramatically. Secondary trauma can cause the displacement of an initially stable, non-displaced rib fracture. When displaced, sharp bony fragments act as internal projectiles that can cause severe secondary internal organ or neurovascular injuries. While parenchymal lung lacerations or intercostal vessel injuries are well-documented, direct penetration of the thoracic aorta by a displaced rib fragment and catastrophic event is exceedingly rare [8]. This report describes a case of a delayed traumatic descending aortic injury caused by the secondary displacement of a left 10th rib fracture following a repeat fall in an elderly patient. It was presumed that the second fall impacted the exact same anatomical site as the initial injury, subsequently driving the bone fragment into the aorta. The patient was successfully treated with emergency thoracic endovascular aortic repair (TEVAR).

## Case presentation

**Figure 1:**
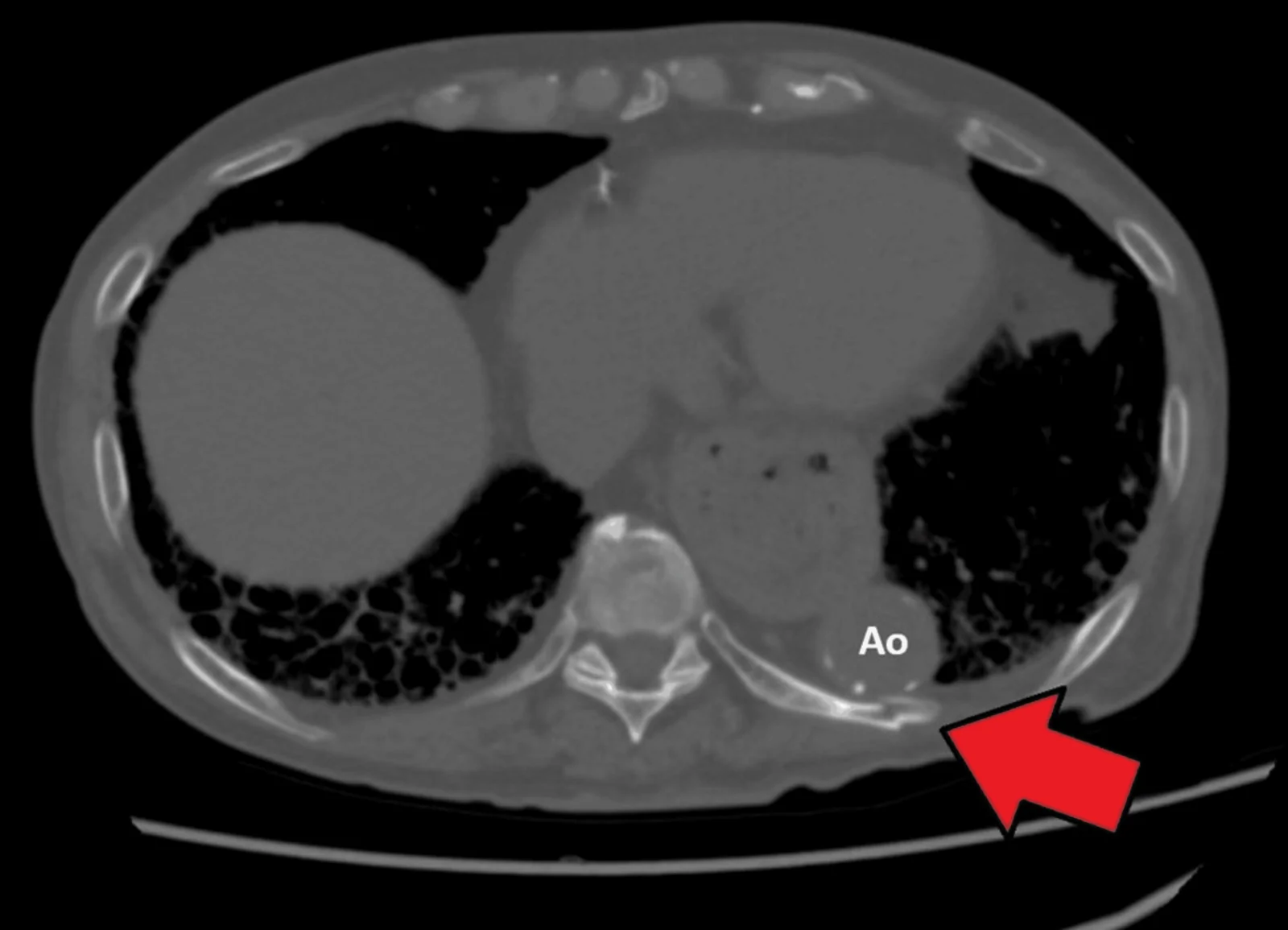
Chest computed tomography obtained after the initial fall. The left 10th rib fracture is evident, but no significant hemothorax indicative of aortic injury is present at this stage. The red arrow indicates the fractured rib, which is located in close proximity to the descending aorta (Ao).

A male in his late 70s with completely independent baseline activities of daily living (ADLs), who was undergoing regular outpatient follow-up at our institution after a history of total aortic arch replacement for an aortic arch aneurysm, presented to the emergency department following a ground-level fall. His chief complaint was isolated left-sided back pain. Upon initial evaluation, his vital signs were stable, and physical examination revealed localized tenderness over the left lower posterior chest wall. A computed tomography (CT) scan demonstrated an acute, non-displaced fracture of the left 10th rib (Figure 1), with no evidence of intrathoracic hemorrhage, pneumothorax, or major vascular injury. Based on his hemodynamic stability and the absence of high-risk imaging findings, he was discharged home with oral analgesics and instructions for conservative outpatient management.

Fifteen days after his initial emergency department visit, the patient experienced another fall inside his home. He was found unresponsive by his family and was emergently transported back to our hospital via ambulance in a state of profound shock. Upon arrival, his vital signs revealed severe hypotension and respiratory distress. Physical examination demonstrated diminished breath sounds over the left hemithorax, and a rapid bedside ultrasound suggested a massive left-sided hemothorax. Despite immediate aggressive fluid resuscitation and the initiation of high-dose vasopressors, his hemodynamic status deteriorated further, leading to a diagnosis of combined obstructive and hemorrhagic shock from a tension hemothorax.

**Figure 2:**
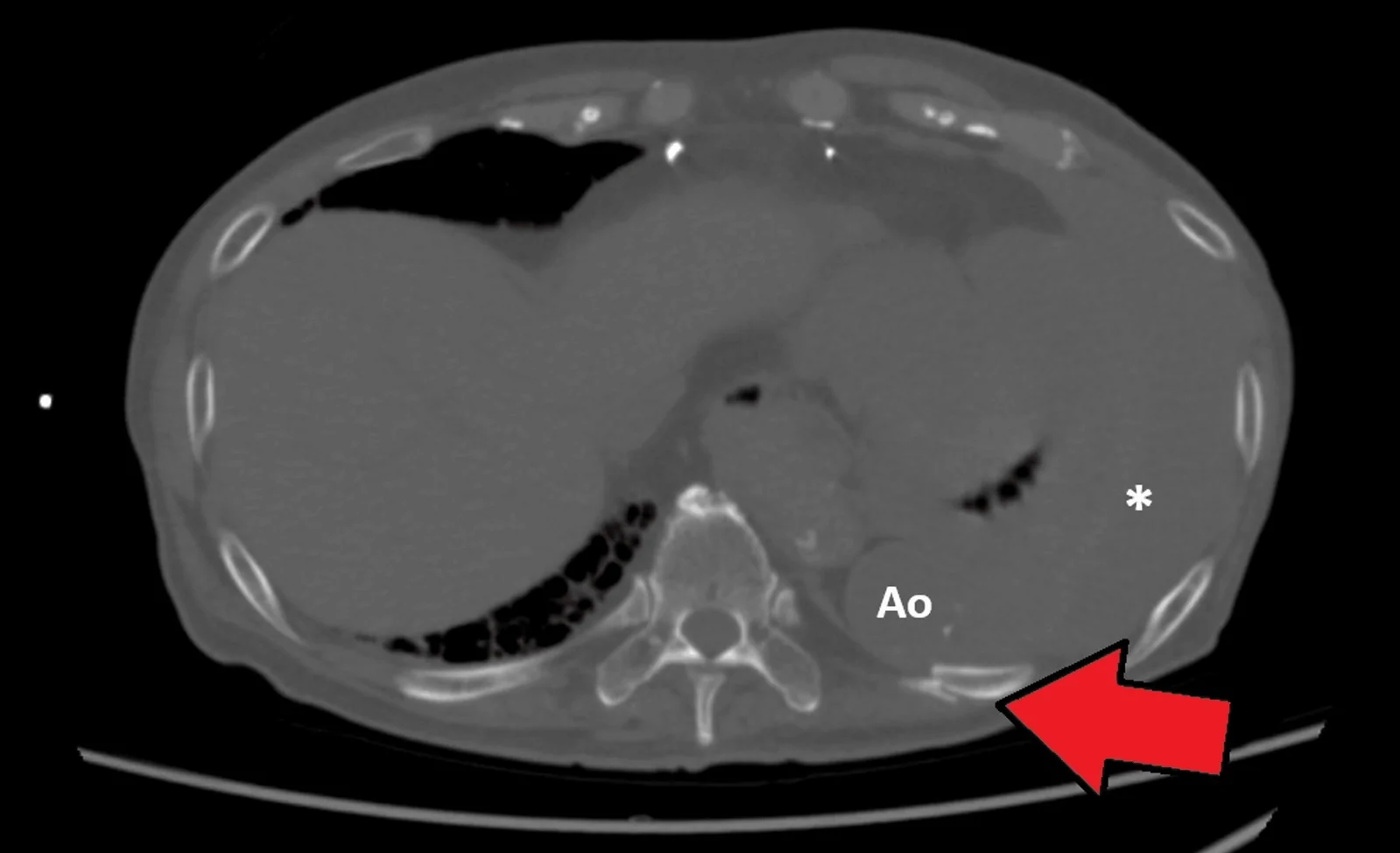
Chest computed tomography obtained after the second fall. Mild displacement of the previously noted left 10th rib fracture is evident, accompanied by a massive left hemothorax. The red arrow indicates the fractured rib, which is located in close proximity to the descending aorta (Ao), and * indicates hemothorax.

An emergency tube thoracostomy was immediately performed in the resuscitation bay, resulting in the evacuation of a massive volume of fresh blood. To temporarily stabilize his collapsing hemodynamics and control the suspected intrathoracic arterial bleeding, an intra-aortic balloon occlusion (IABO) catheter was urgently inserted. Urgent contrast-enhanced angiography was subsequently performed, which revealed active contrast extravasation from the descending thoracic aorta. The site of the aortic laceration corresponded precisely to the location of the previously diagnosed left 10th rib fracture (Figure 2), which had become displaced anteromedially as a direct result of the second fall, penetrating the aortic wall.

**Figure 3:**
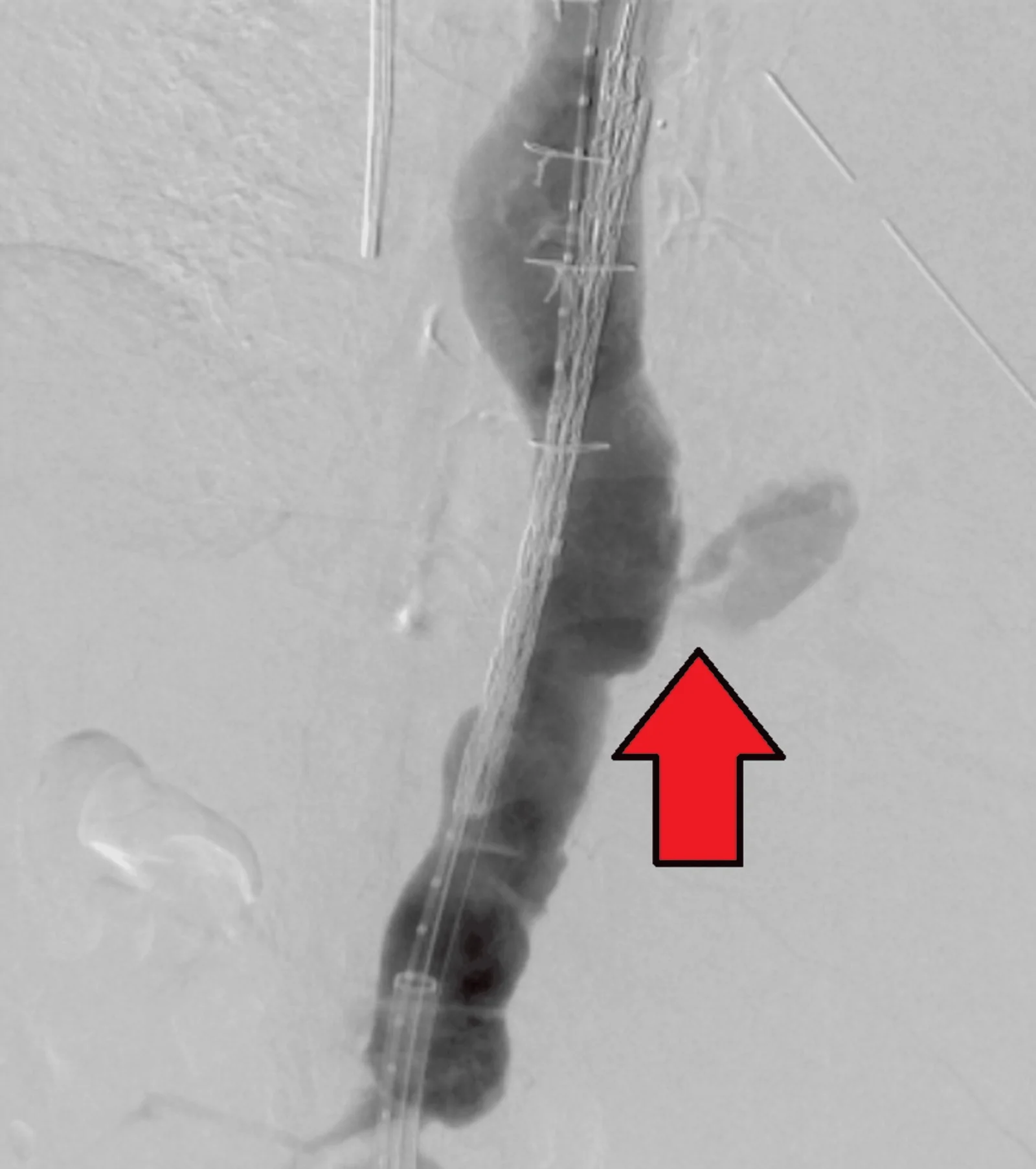
Thoracic aortogram obtained after the second fall. Active contrast extravasation from the aorta is evident at the level of the previously noted rib fractures (red arrow).

**Figure 4:**
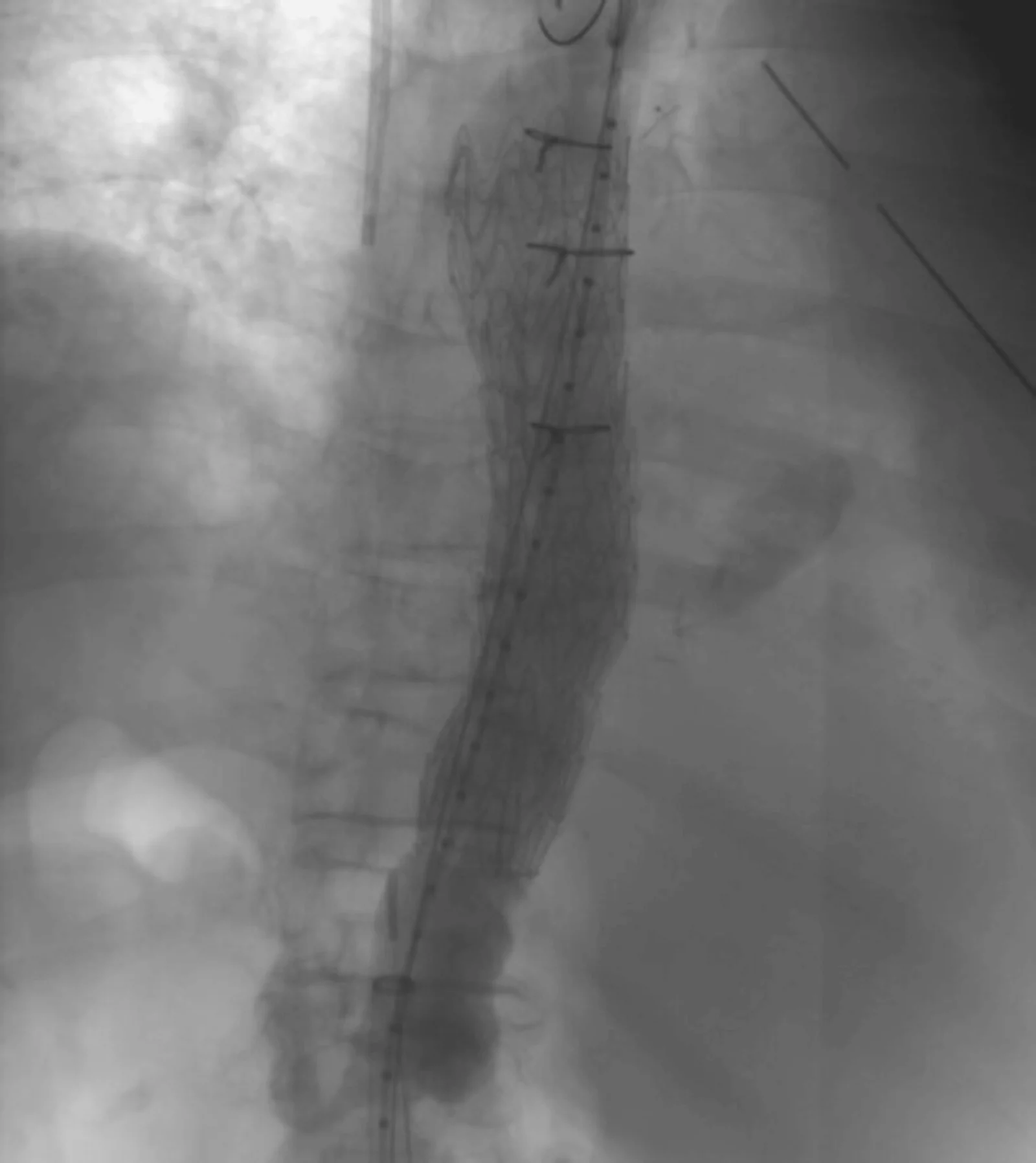
Completion thoracic aortogram following emergency thoracic endovascular aortic repair. The extravasation noted prior to the procedure has completely resolved.

The patient was immediately transferred to the operating suite for emergency TEVAR to exclude the aortic injury. The endovascular stent graft was successfully deployed across the lacerated segment of the descending aorta, achieving complete cessation of the extravasation (Figures 3, 4).

Periprooperatively, the patient required activation of a massive transfusion protocol due to profound hemorrhagic shock and secondary consumptive disseminated intravascular coagulation (DIC). The diagnosis of DIC was established based on the Japanese Association for Acute Medicine (JAAM) DIC scoring criteria [9]. Preoperatively, he received 10 units of red blood cells (RBC) and 12 units of fresh-frozen plasma (FFP). Postoperatively, an additional 14 units of RBC, four units of FFP, and 20 units of platelet concentrates (PC) were administered to correct his coagulopathy.

Following the procedure, the patient was admitted to the intensive care unit (ICU). Further comprehensive evaluations were conducted in the ICU to investigate the etiology of his recurrent falls. However, no obvious underlying medical causes were identified, leading to the conclusion that the injuries were the result of simple, repeated mechanical falls. His postoperative DIC was managed with targeted, regular blood product replacements and supportive care. His vital signs and laboratory parameters gradually stabilized over the subsequent days. He was successfully extubated on postoperative day 11 and was subsequently transferred to the general ward for rehabilitation without any permanent neurological or ischemic sequelae.

## Discussion

Traumatic aortic injury resulting from a rib fracture is an uncommon clinical entity [8], as the thoracic aorta is anatomically protected by the spinal column and surrounding mediastinal structures. Most traumatic aortic injuries are caused by high-energy deceleration mechanisms, such as motor vehicle accidents. Although aortic injuries caused by rib fractures have been previously reported in the literature [8,10], they are extremely rare when considering the overall vast incidence of rib fractures. Furthermore, it is exceptionally rare for such an injury to present as a delayed, life-threatening event [10].

In this patient, the anatomical location of the left 10th rib fracture placed it in close proximity to the lower descending thoracic aorta, rendering the vessel vulnerable to penetration once the rib fragment became severely displaced during the second impact.

The most striking feature of this case is the delayed presentation, occurring 15 days after the initial trauma. This introduces a major clinical pitfall in emergency trauma management. While an initial non-displaced rib fracture is considered benign, it remains a latent hazard in elderly patients who have underlying gait instability or frailty. A secondary fall can easily convert a stable skeletal injury into a sharp, displaced fragment capable of slicing adjacent major vessels. Clinicians must recognize that a patient's initial stability does not guarantee long-term safety if the underlying risk factors for recurrent falls are not addressed.

The successful management of this life-threatening scenario was highly dependent on a coordinated, multi-modal emergency response. The utilization of an IABO catheter provided a crucial hemodynamic bridge, allowing temporary proximal control of the massive hemorrhage while avoiding the high morbidity associated with an emergency open thoracotomy in an unstable elderly patient. Furthermore, TEVAR has emerged as the gold standard for the management of traumatic thoracic aortic injuries, offering a minimally invasive option that avoids the physiological stress of aortic cross-clamping and open surgical reconstruction. In the presence of severe hemorrhagic DIC, the speed and low physiological impact of TEVAR, combined with a vigorous massive transfusion protocol, are critical to surviving what would otherwise be a fatal vascular injury.

## Conclusions

This case serves as a critical reminder that clinicians must never underestimate the potential lethality of simple rib fractures in the elderly. An initially stable, non-displaced rib fracture can become a fatal weapon if a secondary fall causes fragment displacement and subsequent major vascular penetration. When managing elderly trauma patients, emergency physicians and trauma surgeons should maintain a high index of suspicion for delayed complications and prioritize comprehensive fall-prevention strategies and close monitoring rather than focusing solely on initial injury severity.
